# Increased contractile responses to 5-hydroxytryptamine and Angiotensin II in high fat diet fed rat thoracic aorta

**DOI:** 10.1186/1476-511X-3-19

**Published:** 2004-08-02

**Authors:** Srinivas Ghatta, Poduri Ramarao

**Affiliations:** 1 Department of Pharmaceutical Sciences, North Dakota State University, Fargo, North Dakota 58105, USA; 2 Department of Pharmacology and Toxicology, National Institute of Pharmaceutical Education and Research, Sector 67, Phase-X, S.A.S. Nagar 160 062, Punjab, India

## Abstract

**Background:**

Feeding normal rats with high dietary levels of saturated fat leads to pathological conditions, which are quite similar to syndrome X in humans. These conditions such as hypertriglyceridemia, hypercholesterolemia, obesity, and hyperglycemia might induce hypertension through various mechanisms. Metabolic syndrome and the resulting NIDDM represent a major clinical challenge because implementation of treatment strategies is difficult. Vascular abnormalities probably contribute to the etiology of many diabetic complications including nephropathy, neuropathy, retinopathy, and cardiomyopathy. It has been shown that in Streptozotocin induced diabetic animals there is an increase in maximal responses to 5-Hydroxytryptamine and Angiotensin II. The purpose of this study was to evaluate High fat diet fed rats for the development of hypertriglyceridemia, hypercholesterolemia, hyperinsulinemia and hyperglycemia and to assess their vascular responses to 5-Hydroxytryptamine and Angiotensin II.

**Methods:**

Male Sprague Dawley rats were used for this study and were divided into two equal groups. One of the groups was fed with normal pellet diet and they served as the control group, whereas the other group was on a high fat diet for 4 weeks. Body weight, plasma triglycerides, plasma cholesterol, and plasma glucose were measured every week. Intraperitoneal glucose tolerance test was performed after 4 weeks of feeding. At the end of fourth week of high fat diet feeding, thoracic aortae were removed, and cut into helical strips for vascular reactivity studies. Dose-response curves of 5-Hydroxytryptamine and Angiotensin II were obtained.

**Results:**

There was no significant difference in pD_2_, with 5-Hydroxytryptamine and Angiotensin II in both groups but E_max _was increased.

**Conclusions:**

These results suggest that hypertension in high fat diet rats is associated with increased in vitro vascular reactivity to 5-HT and Ang II.

## Background

Syndrome X comprises a plethora of conditions such as obesity, dyslipidemia, impaired glucose tolerance, insulin resistance and hypertension [1]. It places stress on multiple organ systems and plays a significant role in the development of other related cardiovascular disorders. Western style diet, which contains high levels of fat, is also considered to be one of the main factors in the development of obesity and insulin resistance. The most common reason for the development of hyperinsulinemia (decreased hepatic insulin clearance and/or increased insulin secretion) from insulin resistance is obesity. Excess fat deposits in the white adipose tissue affects insulin mediated glucose metabolism in non adipose tissues, causes disordered insulin response and increases lipid deposition [2]. However, pathophysiologies of vascular complications in syndrome X have not been fully understood. 5-hydroxytryptamine (5-HT) is shown to be related to pathogenesis of vasculopathy in diabetes. Various studies in humans and rabbits have reported increased plasma 5-HT levels and enhanced contraction to 5-HT in diabetes mellitus [3-5]. Another vasopressor peptide, Angiotensin II (Ang II) is involved in cardiovascular complications of diabetes mellitus. Many have reported that the vascular Renin angiotensin system is one of the key systems in the etiology of vascular alterations in early stages of diabetes. It is already established that 5-HT and Ang II responses are altered in the aortic rings of streptozotocin (STZ) induced diabetic rats [6,7] and high fructose diet fed rats [8]. However, very little information is available in the literature regarding the vascular contractile responses to vasopressor agents such as 5-HT and Ang II in HFD fed rats.

The aim of this study was to elucidate the contractile responses to 5-HT and Ang II in HFD fed rat thoracic aorta which will provide an avenue for further exploratory studies. We have selected HFD fed rat model for our study because it is a useful model of the putative effects of excess fat intake in humans and it represents the major sub type of diabetes mellitus, non insulin dependent diabetes mellitus (NIDDM).

## Results

### Biochemical measurements

**Figure 1:**
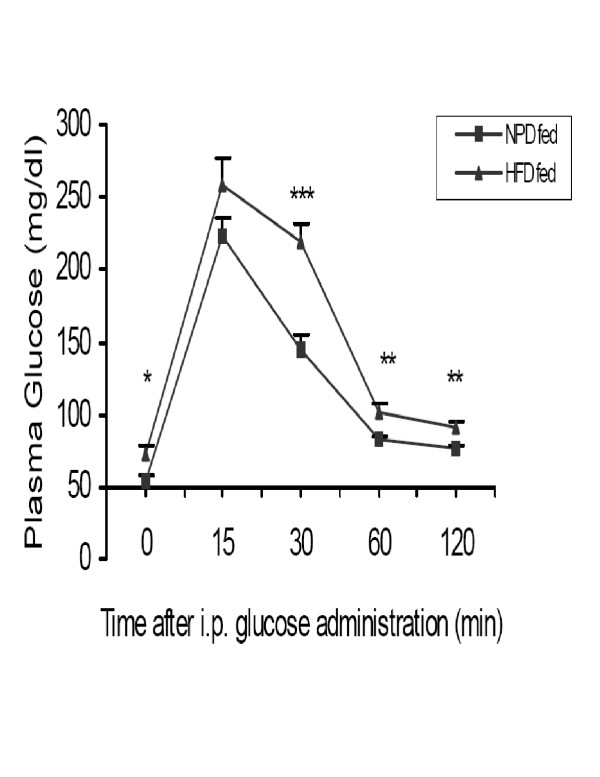
Effect of HFD on intra peritoneal glucose tolerance test (IPGTT) in rats, as compared to NPD fed group. All values are expressed as mean ± SEM (n = 8) *p < 0.05, **p < 0.01, ***p < 0.001 Vs NPD fed group

HFD fed rats showed significant increase in body weight as compared to NPD fed rats (Table 2). In addition, the rats fed HFD showed significant elevation in the basal plasma glucose, triglyceride, total cholesterol and insulin levels at the end of four weeks of dietary manipulation as compared to NPD fed control groups (Table 2). The HFD fed rats exhibited significant elevation in basal fasting glucose and showed significant impairment in glucose tolerance to exogenously administered glucose (Fig 1). Estimation of AUC values indicated 28.4 % increase in plasma glucose of HFD fed when compared to NPD fed rats.

**Table 2:** Various parameters of NPD and HFD fed rats

**Parameter**	**NPD fed**	**HFD fed**
**Body weight (gm)**	259.2 ± 5.3	315.0 ± 6.5***
**plasma glucose (mg/dl)**	87.5 ± 2.5	111.0 ± 0.9***
**Plasma triglycerides (mg/dl)**	41.3 ± 1.7	89.7 ± 7.2***
**Plasma cholesterol (mg/dl)**	48.9 ± 3.7	87.5 ± 2.6***
**Plasma Insulin (ng/ml)**	1.90 ± 0.15	3.05 ± 0.21*
**pD_2 _of 5-HT**	5.74 ± 0.09	6.00 ± 0.06
**E_max _of 5-HT**	30.0 ± 1.8	49.0 ± 2.0**
**pD_2 _of Ang II**	7.69 ± 0.08	7.31 ± 0.18
**E_max _of Ang II**	25.0 ± 3.4	34.0 ± 3.0*

Each point is represented as mean ± SEM *p < 0.05, **p < 0.01 and ***p < 0.001 Vs NPD fed group

### Vascular Responses

**Figure 2:**
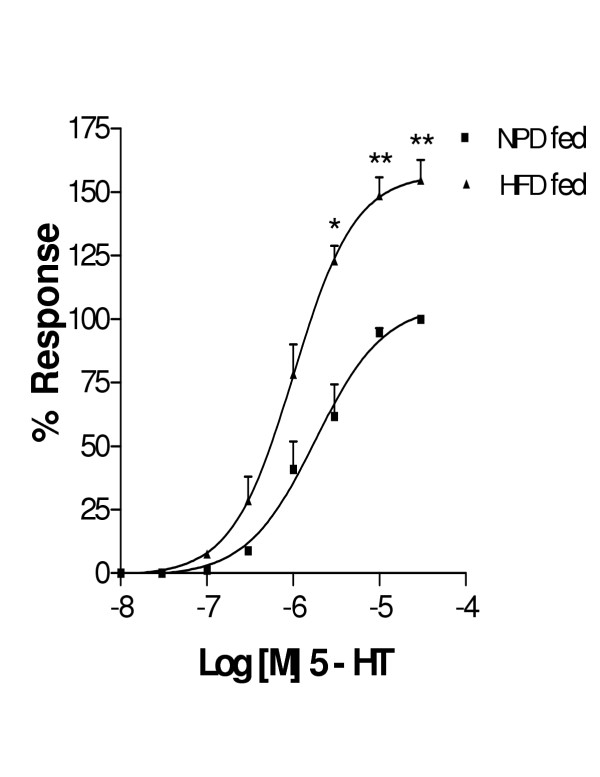
Cumulative concentration response curve to 5-HT in helically cut aortic strip preparations obtained from NPD fed and HFD fed rats. Each point is represented as mean ± SEM (n = 5) *p < 0.05, **p < 0.01 Vs NPD fed group

**Figure 3:**
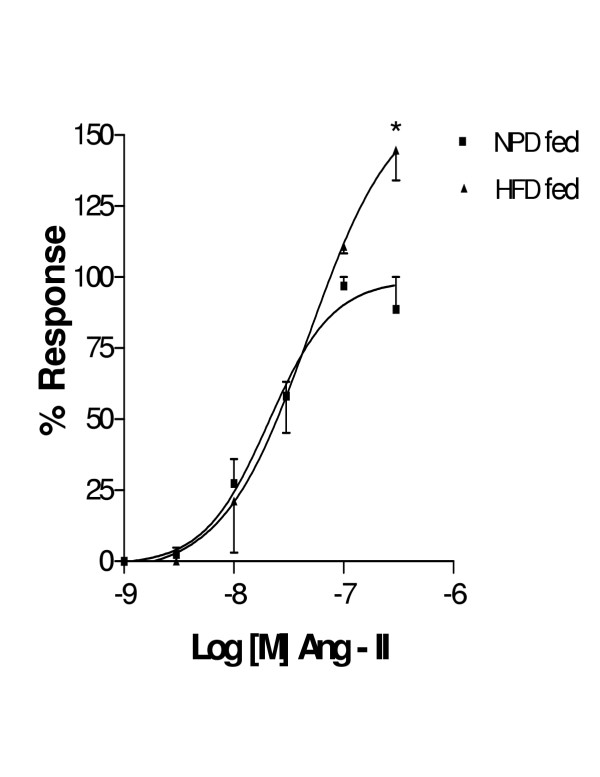
Cumulative concentration response curve to Ang II in helically cut aortic strip preparations obtained from NPD fed and HFD fed rats. Each point is represented as mean ± SEM (n = 5) *p < 0.05, **p < 0.01 Vs NPD fed group

Cumulative concentration response curves of 5-HT and Ang II for both HFD fed rat thoracic aortae showed an increase in E_max _(Fig 2 and 3) with out any change in pD_2 _values when compared to NPD fed rats (Table 2). There was no significant change in both E_max _and pD_2 _values with KCl in both groups (data not shown). The order of potency of agonists in both groups was Ang II>5-HT>KCl.

## Discussion

Obesity is a major risk factor for several metabolic diseases, frequently clustering to form the metabolic syndrome or syndrome X [9]. Obese people have increased incidence of NIDDM with high percentage of mortality and morbidity [10]. Western style diet, which is abundant with calorically dense and saturated fatty foods, is considered to be the main factor in the development of obesity and insulin resistance. Our studies have shown that HFD causes increase in bodyweight when compared to NPD after four weeks of dietary manipulation in rats.

Hyperglycemia is observed in insulin resistance where glucose utilization is reduced. We saw significant elevations in blood glucose levels. Intraperitoneal glucose tolerance tests confirm severe glucose intolerance. Oversupply of dietary lipids causes insulin resistance in rats [11]. Randle glucose fatty acid cycle suggested that the body prefers excess lipid stores to glucose for metabolic oxidation in insulin resistance [12]. Our HFD model also exhibited high plasma triglyceride levels. Hence studies on our experimental model in compliance with Randle et al findings suggested that HFD feeding causes insulin resistance [13].

Insulin resistance with compensatory hyperinsulinemia is a prominent feature of metabolic syndrome. The most common reason for the development of hyperinsulinemia in insulin resistance is obesity. It stands as one of the major cardiovascular risk factors in patients with obesity. The present study on HFD rats demonstrated higher plasma insulin levels than control values. This marked hyperinsulinemia could be due to a combination of increased β-cell mass and decreased insulin clearance, as well as failure of insulin to suppress hepatic gluconeogenesis [14].

Elevated cholesterol is also observed in insulin resistant individuals. For this reason we measured plasma cholesterol levels which were found to be more than normal values. Previous studies have reported a down regulation of LDL receptors and associated decrease in LDL clearance, increased total cholesterol levels [15].

According to National Cholesterol Education Program's Adult Treatment Panel III (Third report) easily measured clinical findings for syndrome X includes increased abdominal circumference, elevated triglycerides, low high-density lipoprotein-cholesterol, and elevated fasting blood glucose and/or elevated blood pressure. Three of these five are required for diagnosis. Our study demonstrated three of the clinical parameters indicating conditions of syndrome X in HFD fed rats [16]. Insulin resistance along with other conditions of syndrome X might induce hypertension by a host of mechanisms involving insulin itself, increased sodium reabsorption and/or enhanced intra cellular concentration of free calcium in vascular smooth muscle [17]. Although the etiology of vascular disorders in metabolic syndrome has not completely been revealed, it is suggested that alterations in the reactivity of blood vessels to neurotransmitters and circulating hormones are responsible for the functional abnormalities of blood vessels.

In order to elaborate the pathways that connect syndrome X to hypertension we have studied the contractile responses to 5-HT and Ang II in both HFD and NPD fed rat thoracic aortae. Previously we have demonstrated increased contractile responses with synthetic alpha adrenoceptor agonist, phenylephrine, in HFD fed rat thoracic aorta [18]. This enabled us to explore the role of these endogenous mediators in the same animal model.

The present vascular studies demonstrated that the magnitude of responses to 5-HT and Ang II was significantly enhanced in HFD fed animals without change in pD_2 _value. Endothelial denudation obviates any related mechanisms such as impairment of NO release, increased destruction of EDRF and substrate availability for the production of EDRF. Hence, the probable reasons for these enhanced 5-HT and Ang II responses may be due to receptor mediated or non-receptor mediated pathways.

The role of non-receptor mediated contraction can be ruled out for there was no change in contractile response to KCl. Vascular studies have also shown functional evidence that hypertension developed in HFD fed rats may be associated with enhanced vasoreactivity to various vasoconstrictor agents. Further the enhanced responses to 5-HT in HFD fed rats could be due to increased PKC as previously reported with STZ and alloxan (ALL) induced diabetic animals [19]. Increased contractions to 5-HT can also be related to 5-HT_2A _upregulation as observed in spontaneously hypertensive rats or due to serotonin acting through alpha adrenoceptors [20].

**Figure 4:**
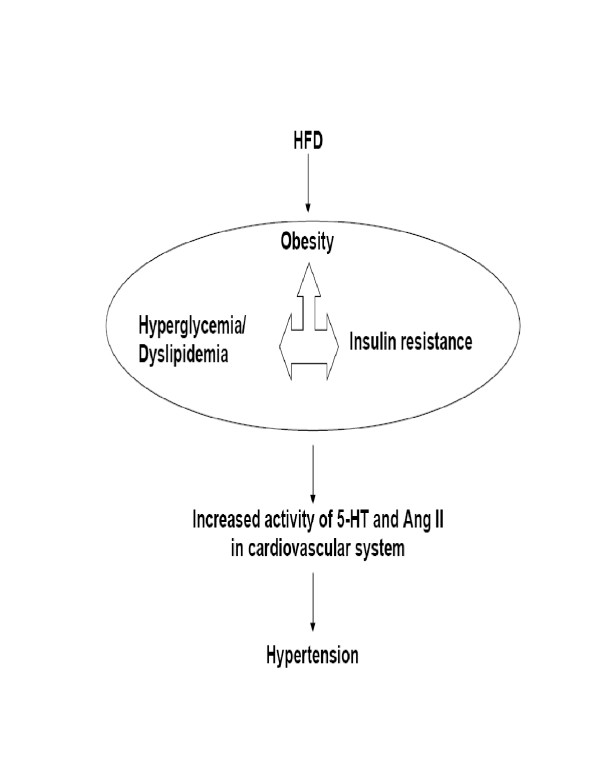
Proposed events underlying syndrome X in HFD rats. HFD leads to condition, in rats, similar to syndrome X. This includes obesity, insulin resistance, hyperglycemia and dyslipidemia which all are interrelated. These events are the initial steps in the cascade towards hypertension. This could be mediated via increased contractile responses to various endogenous mediators such as 5-HT and Ang II.

Increased Ang II responses in HFD fed rats are may be due to upregulation of Ang II receptors as observed in hyperinsulinemia or via amplified secondary messenger systems [21]. A proposed scheme of events is given in Fig 4.

In summary, the present study has shown that HFD feeding in rats produces conditions similar to syndrome X. Increased vasocontractile responses observed in the model are not only mediated via alpha adrenoceptors but also due to 5-HT and Ang II (see Figure 4).

More robust studies on the secondary messenger systems of 5-HT and Ang II will provide valuable insights into the mechanisms underlying increased vascular contractility in insulin resistance.

## Materials and Methods

### Tissue Preparation

Male Sprague-Dawley rats (central animal facility, National Institute of Pharmaceutical Education and Research (NIPER), India), 160–200 g, were kept in controlled environmental conditions with room temperature 22 ± 2°C, humidity 55 ± 5% and 12-h light/dark cycles. All the animals had free access to food and water. The rats were divided into two dietary groups and fed with standard rat normal pellet diet (NPD) (3.8 kcal/g, carbohydrate 67%, protein 21%, fat 12% kcal) and HFD (5.3 kcal/g, carbohydrate 17%, protein 25%, fat 58% kcal). Composition of HFD is described in Table 1.

**Table 1:** Composition of HFD

	(g)
Powdered pellet diet	364
Lard	310
Casein	250
Cholesterol	10
DL-Methionine	3
Yee-sac powder	1
Vitamin and mineral mix powder	60
Sodium chloride	2

#### Biochemical measurements

Blood samples from the retro orbital plexus of anaesthetized rats (Pentobarbitone 45 mg/kg, i.p.,) were collected into the heparinized tubes and immediately centrifuged at 5000 rpm for the separation of plasma. Plasma was stored at -20°C until assayed. The plasma was used for the estimation of glucose (Qualigens, Mumbai, India), triglycerides, and cholesterol (Chema diagnostica, Jesi, Italy) by commercial kits. Plasma insulin was determined by radioimmuno assay using rat insulin as standard (Linco research, St. Charles, MO, USA)

#### Intra peritoneal glucose tolerance test (IPGTT)

Glucose tolerance tests were carried out after four weeks of feeding of both NPD and HFD. After an overnight fast, blood samples were collected from the retro orbital plexus. Glucose levels were measured at time zero (0 min) and glucose was injected into the rats (2 g/Kg/4 ml, i.p.,). Additional blood samples were taken at 15, 30, 60 and 120 min. following the glucose load. Plasma glucose levels were measured by the glucose oxidase reaction (GOD/POD) using commercial kit (Qualigens, Mumbai, India). Area under the curve (AUC) was calculated for both NPD and HFD fed rats.

#### Vascular Studies

After 4 weeks of feeding, rats were sacrificed by cervical dislocation. The section of the aorta from between the aortic arch and the diaphragm was removed from the euthanized rats and placed in oxygenated, modified Krebs-Henseleit solution (KHS; in mM: 118 NaCl, 4.7 KCl, 25 NaHCO_3_, 2.6 CaCl_2 _2H_2_O 1.2 NaH_2_PO_4_, 1.2 MgCl_2 _6H_2_O, 5.5 glucose). With the help of a steel rod, aortic endothelium was deliberately denuded. The aorta was cut into helical strips 3 mm wide, 20 mm long and then placed in a well-oxygenated (95% O_2_-5% CO_2_) bath of 10-ml KHS with one end connected to a tissue holder and the other to an isotonic transducer (Bio Devices, Ambala, India). The tissue was equilibrated for 60 min under a resting tension of 1.0 g. At the beginning of each experiment, aortic strips were primed with depolarizing concentration (90 mM) potassium chloride (KCl). After the equilibration period, contractile responses to various concentrations of 5-HT (10 nM-30 μM) and Ang II (1 nM-300 nM) were recorded.

### Data analysis

Contraction responses are expressed as percentage. For each contractile agent, both the maximal contraction (E_max_) and the concentration necessary to produce 50% of its maximal response (EC_50_) were determined. The EC_50 _values were converted to the negative logarithms and expressed as pD_2_. Results were shown as mean ± SEM; n refers to the number of animals from which vessels were taken. Agonist potencies and maximal effects were compared by student's *t *test by using statistical software (GraphPad Prism 3.01). Values were considered significantly different at p < 0.05.

### Drugs and solutions

The following drugs were used in this study: 5-HT (RBI, USA), Ang II (Bachem, Switzerland), All drugs were dissolved in KHS. Drugs were added to the organ chambers in volumes not greater than 0.2 ml.

## List of abbreviations

HFD – High fat diet

NPD – Normal pellet diet

NIDDM – Non insulin dependent diabetes mellitus

STZ – Streptozotocin

5-HT – 5-Hydroxytryptamine

Ang II – Angiotensin II

KHS – Krebs-Henseleit solution

GOD/POD – Glucose oxidase/peroxidase

AUC – area under the curve

E_max _– Maximal response

EC_50 _– Concentration required producing 50% of maximal response

pD_2 _– -log EC_50_

## Authors' contributions

SG carried out the all experimentations. PR has conceived the study and participated in its design and coordination. Authors read and approved the final manuscript.
